# Supplementary material for: Antimicrobial Peptides Originating from Expression Libraries of Aurelia aurita and Mnemiopsis leidyi Prevent Biofilm Formation of Opportunistic Pathogens
Source: Microorganisms. 2023 Aug 29;11(9):2184. doi: 10.3390/microorganisms11092184 (PMC10537229; doi:10.3390/microorganisms11092184)
Supplement: Supplementary file 1 [file microorganisms-11-02184-s001.zip › microorganisms-2486178-supplementary.pdf]

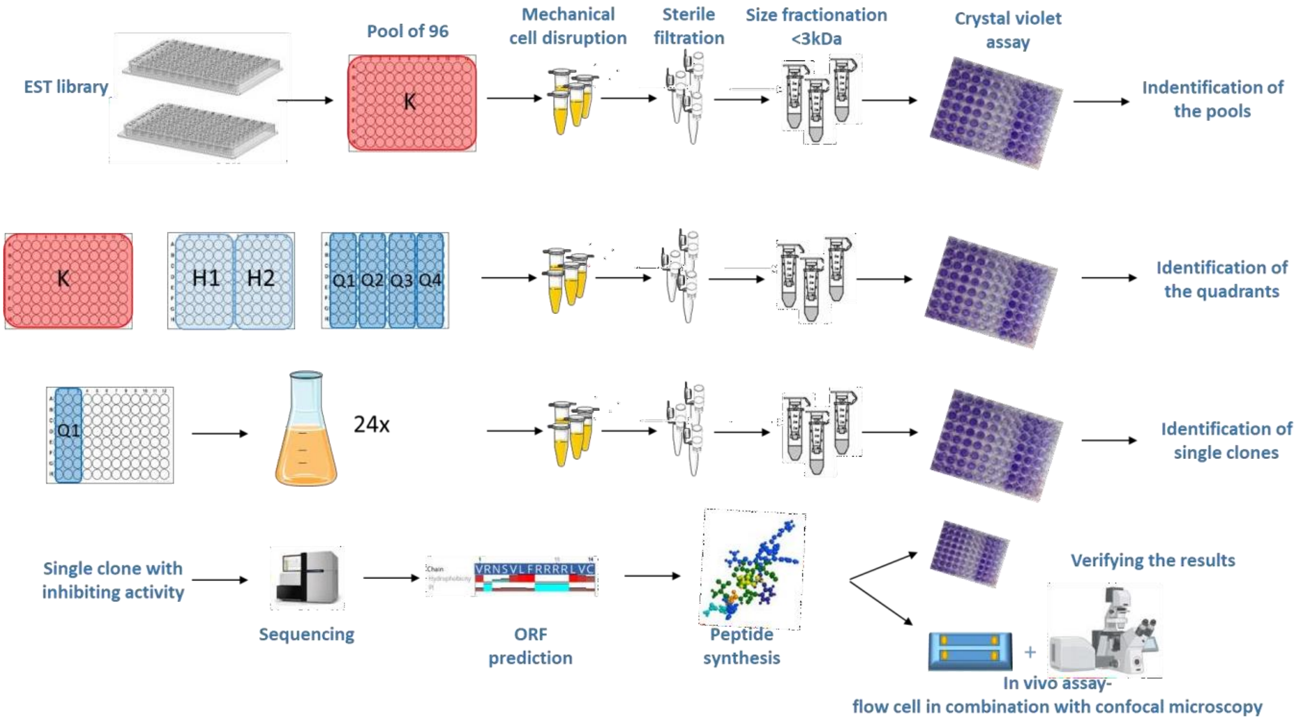

Figure S1: Identification of biofilm-inhibiting clones from the cDNA expression library using the crystal violet assay. Cell-free size-fractionated cell extracts were prepared successively from pools of 96, 48, 24, and single clones. Biofilm-inhibiting single clones were further characterized toidentify the corresponding peptides.

Table S1: Identified biofilm-preventing cDNA single clones. Biofilm-preventing single clones were identified in cDNA expression libraries of *A. aurita* and *M. leidyi* using the crystal violet assay. Activity-conferring sequences were gainedand the respective sORFs N-terminally fused to the vector-derived Histidine-tag were translated into peptide sequences. NCBI BLASTp results for those peptide sequences are shown.

Single clone designation	Peptide designation	Insert (5'-3') (vector proportion)	Amino acid sequences of the in-frame ORF	NCBI BLASTp			
				Best homologue	Assession No.	Query Coverage [%]	Identity (%)
Aa_112_4H	BiP_Aa_1	atgcatcatcatcatcatcacatcacaagtttgtacaaaaaagttggcc<br>gccgagtacgatgaaaccgaatctgaagaggaaccatccgaagtagaat<br>cagctgatgatgaaattgaaggtgttgcagcgaataaaggttccgatcc<br>tttattctggcgacgccgaagccgccgccgtattgtctaccgtcgtcgt<br>tggtatacccgtcgtcgttgctactacgctcgccgccgatacatcgctc<br>gccgccgatacatcgctcgcagaagatgctatgctttaataacacgcag<br>aagatggttcagacgaagaggttaacttcccatggcacgaaaattgctg<br>aagacattcgttgattaatcgtagagctgtaaagggtgtttaaaaattg<br>tagtagatgctgattctgattttacgctgataggtgcatgc	RRVR	no significant similarity			
Aa_112_6C	BiP_Aa_2	atgcatcatcatcatcatcacatcacaagtttgtacaaaaaagttggcc<br>aacttttttgtacaaagttgtcccccacatag	QLFCTKLSPT	heat shock protein Hsp110 [*Aurelia aurita*]	AAX09921.2	70	71
Aa_127_8A	BiP_Aa_3	atgcatcatcatcatcatcacatcacaagtttgtacaaaaaagttggat<br>ggtcttgagcagtacacagaagtaaaatcagtcatcatcaaaatggacc<br>agaagaatgcttaaagagaccagaacttcaagacaataataaattcatc<br>atgcatatttcatcgttcatcataaatctcgattgtagtttgttgaagg<br>tgccttcttaactgaatgtgtccaactagctaaaaaaaaaaattcctag ctttttaa	WS	no significant similarity			
Aa_127_8E	BiP_Aa_4	atgcatcatcatcatcatcacatcacaagtttgtacaaaaaagttggca<br>cacattctacaatgaacttcgagttgcaccagaggagcatccagtcctg<br>ctcaccgaagctcctttaaatccaaaagctaacagggaaaagatgacac<br>aaattatgttcgaaaccttcaacagccctgcaatgtacgtcgccatcca<br>agccgtactgtccctgtacgcctctggtcgtaccaccggtatcgttctt<br>gattccggagatggtgtcagccacactgtcccaatctacgaaggttatg<br>cccttccccacgccatcatccgtttggatttggctggacgtgatttgac<br>cgactacttgatgaagatcctcaccgagagaggttactcattcaccacc<br>accgccgaaagggaaatcgtcagagacatcaaggagaaactctgctatg<br>tcgcactcgacttccaacaagaaatgctcacagcatcaaccagctcaag<br>cttggagaagagctacgaattacctgacgggacaggtcatcaccatcgg<br>aaacgagagattcaggtgcccagaaacccctcttctaacccgcattcat<br>cggaatggaatcaagcggaatccacgagaccaccatacaaactcaatca<br>tgaaaatgcgatgtcgacatccgtaggacttgcatgccaacaccgtctt<br>tgtctggaggtacgactatgttccccaggtatccgccgacagaatgcac<br>aaggagatcgcttccctcgcacccctcaaccatgaaaattcagatcatc<br>gccccaccagagtaggaaactactcccgtatgggatcggaggctccatc<br>ttggcttcccctctccacctctccaccagatgtcgcatctcgcaatcaa<br>gaatatggatgaatcctgggcccatccattctctcaccacgaaaacgct<br>tccttacaccgcctctgcgcacacttttcaa	THSTMNFELHQRSIQSCSPKLL	retinoid X receptor [*Aurelia aurita*]	AGT42223.1	40	71
Aa_127_8F	BiP_Aa_5	atgcatcatcatcatcatcacatcacaagtttgtacaaaaaagttgggg<br>taaggaacagtgttctattccgtcgcaggcgacttgtatgttagaaagt<br>agtagcactgttttagtagtttaacgataaatcttgaaatgatatctaa caaaatgtgttga	VRNSVLFRRRRLVC	hypothetical protein [*Aurelia aurita*]	AGN03863.1	35	100
Aa_127_8H	BiP_Aa_6	atgcatcatcatcatcatcacatcacaagtttgtacaaaaaagttggga<br>caaactttacaaggaagttcccagttataaactcatcaccccatcagtg<br>gtatctgaaagattgaaggtcagagtttcactcgcacgcacatgcttga<br>aagaactgttgggaaaaggccttatccgggaagtttccaaacatagtga<br>tcagatgatctacactagagctacaaaagaggctgccaaataactttgt<br>ttataagcaatgttgaatcttgaaaatgagtaatctttaaaagcta	TNFTRKFPVINSSPHQWYLKD	toxin TX1 [*Aurelia aurita*]	AFK76348.1	66	55

Ml_068_11H	BiP_Ml_1	atgcatcatcatcatcatcacatcacaagtttgtacaaaaaagttggaa<br>taatttgagcaataagcgagcttcgatgaaagagagcagaaagaggtag<br>tatcgggggacaaacgaactgtccttaaagcgtaacagactacagaata<br>cacccatcgtgcgttcaggatttttggcaagttcatgtgacagtaaaac<br>gagatgagttcacgagaggtttgatctcaaggagcaactgcgagctata<br>cgacaacgtatacaagtacaaatacgatctccatcaatcagcaccgttc<br>aaatatttggtataaattaatgcagccgggattttgttttgcttccttt<br>ctatttcatagctttgaacaaatacgagaccatggtactcggcgttgtg<br>tactttgattaataataatagtcatagtttgaattatttaaactttcgt<br>agtcttagtctccgcctgtcccattaaagattcttaaatcgctgttgtg<br>actgcgaactttcgcccatttttaacttgtatgcctcaatttttcaaat<br>tcacttcttatgttttaattaatacagttgagaattatgtggctacgag<br>catcagttggagcactgaagctaatatttgttacccggactgtcatact<br>tataggtctcctcatctccataaaaaattaaaaaacaaatggagtactg<br>ttccaattaattttctactattttattgagttttaaaattactttattt actac	II	no significant similarity			
Ml_011_11H	BiP_Ml_2	atgcatcatcatcatcatcacatcacaagtttgtacaaaaaagttggca<br>actactattaattaaataatttgaggcaaggctccaggttcttgcgtaa<br>gcggcattcaagatggcgcccaaacacaacaatatggtttttgacagcc<br>atttccacaaggattggcaaaggtatgtcaaaacctggttcaaccaagc<br>cggtaagaagaagcgcaggcgcacaaaacgtatggagaaggctaaagct<br>gtcgcccctcgcccagttgctggtctgctccgacctgttgtacattgtc<br>agactatcagatacaacgcccgagtcagagccggccgaggttttaccct<br>cgacgaactcaaggctgctggtatcaacaagaagcaagctctctcaatc<br>ggaattgctgttgaccacagaaggaagaacaggtctcaggagagtctcc<br>aagctaatgttctcagactgaaggagtacaagagcaggctcatcctctt<br>cccacgaaaggcctccaaacccaagaagggagacagcacccaggctgag<br>atcgatgtcgccactcagctgaccggacctgtcctacccatcaaacaga<br>cctggtctgataccaccgccagagcaatcacggacgaggagaagaaaca<br>atctgctttccagacgatgagaatgtacagagccaatgttcgacttgtt<br>ggagttcgagaaaaacgagccaaggaagccgccgaggatgacggtctgg<br>gagtcaagaagaagaagaaaataatttatttcggttttttatctttta tcgataatgtca	NYY	no significant similarity			
Ml_010_9A	BiP_Ml_3	atgcatcatcatcatcatcacatcacaagtttgtacaaaaaagttggcg<br>gccgcacaactttgtacaagaaagttgggttttttttttttttaaca<br>tttttaaattttattatttctattttgatttattttatatgacaaatta<br>atttattttcccatttaatctaattgttaaaaaaaaacaaaaaaaaaaa<br>ccaaaaacaaaaaaccccccacctttcttgtccaaattggtgatctagg<br>tatattcggatccggttgctaccaaaccccgaaaggaacttgccttggc<br>tgcccccccgctgcccaataactagcataaccccttggggccttcaaa<br>cgggttttgagggggtttttgctgaaaggaggaacaattcccggttttc ccga	GRTTLYKKVGFFFFF	ND5 gene product [Mnemiopsis leidyi]	YP_004927440.1	73	100

Table S2: Statistics derived from unpaired t-test for growth-inhibiting effects of peptides shown in Figure 2. Unpaired t-test results were calculated with GraphPad. SD, standard deviation calculated from 2 biological, each with 8 technical replicates; t; t-statistic; df, degrees of freedom

K. oxytoca

treatment	control	IDR-1018	BiP_Aa_2	BiP_Aa_4	BiP_Aa_5	BiP_Aa_6	BiP_Ml_3
turbidity	1.21326667	1.186925	1.2036875	1.219475	1.25715	1.2508	1.25545
SD	0.02265977	0.01371174	0.01282045	0.05077339	0.01444717	0.02106066	0.02517361
replicates	16	16	16	16	16	16	16
p-value		0.0004	0.1515	0.6583	0.0001	0.0001	0.0001
t		3.9783	1.4717	0.4466	6.5318	4.8531	4.9818
df		30	30	30	30	30	30

Symbol	Meaning
	growth inhibition
	growth promotion
ns	
*	P > 0.05
**	P ≤ 0.05
***	P ≤ 0.01
	P ≤ 0.001

P.aeruginosa

treatment	control	IDR-1018	BiP_Aa_2	BiP_Aa_4	BiP_Aa_5	BiP_Aa_6	BiP_Ml_3
turbidity	1.2788369	1.2296125	1.2593	1.28101429	1.24941429	1.2841125	1.254575
SD	0.02236065	0.03617095	0.0265339	0.03674385	0.0347689	0.03765816	0.02866537
replicates	16	16	16	16	16	16	16
p-value		0.0001	0.0318	0.8409	0.0079	0.6334	0.0121
t		4.6302	2.2521	0.2025	2.847	0.4818	2.6694
df		30	30	30	30	30	30

S. aureus

treatment	control	IDR-1018	BiP_Aa_2	BiP_Aa_4	BiP_Aa_5	BiP_Aa_6	BiP_Ml_3
turbidity	1.37192917	1.0359875	1.3037125	1.346525	1.3122125	1.379525	1.34535
SD	0.03806921	0.17578437	0.02387994	0.03444217	0.03913066	0.05216267	0.02014105
replicates	16	16	16	16	16	16	16
p-value		0.0001	0.0001	0.057	0.0001	0.6414	0.0195
t		7.4712	6.0719	1.9794	4.3754	0.4705	2.4685
df		30	30	30	30	30	30

S. epidermidis

treatment	control	IDR-1018	BiP_Aa_2	BiP_Aa_4	BiP_Aa_5	BiP_Aa_6	BiP_Ml_3
turbidity	1.3398	1.2033875	1.36845	1.3859125	1.3013125	1.39345	1.36715
SD	0.01995141	0.01700263	0.01306823	0.0152605	0.02536809	0.01	0.02
replicates	16	16	16	16	16	16	16
p-value		0.0001	0.0001	0.0001	0.0001	0.0001	0.0005
t		20.8156	4.805	7.3432	4.7701	9.6159	3.8726
df		30	30	30	30	30	30
